# Psychoactive drugs and false memory: comparison of dextroamphetamine and delta-9-tetrahydrocannabinol on false recognition

**DOI:** 10.1007/s00213-011-2374-5

**Published:** 2011-06-07

**Authors:** Michael E. Ballard, David A. Gallo, Harriet de Wit

**Affiliations:** Department of Psychiatry and Behavioral Neuroscience, University of Chicago, 5841 S. Maryland Ave., MC3077, Chicago, IL 60637, USA, mballard@uchicago.edu; Department of Psychology, University of Chicago, 5848 S. University Ave., Chicago, IL 60637, USA; Department of Psychiatry and Behavioral Neuroscience, University of Chicago, 5841 S. Maryland Ave., MC3077, Chicago, IL 60637, USA

**Keywords:** Amphetamine, Cannabinoids, Learning and memory, Recognition, Human, Psychostimulant, Object recognition, Learning

## Abstract

**Rationale:**

Several psychoactive drugs are known to influence episodic memory. However, these drugs’ effects on false memory, or the tendency to incorrectly remember nonstudied information, remain poorly understood.

**Objectives:**

Here, we examined the effects of two commonly used psychoactive drugs, one with memory-enhancing properties (dextroamphetamine; AMP), and another with memory-impairing properties (Δ^9^-tetrahydrocannabinol; THC), on false memory using the Deese/Roediger–McDermott (DRM) illusion.

**Methods:**

Two parallel studies were conducted in which healthy volunteers received either AMP (0, 10, and 20 mg) or THC (0, 7.5, and 15 mg) in within-subjects, randomized, double-blind designs. Participants studied DRM word lists under the influence of the drugs, and their recognition memory for the studied words was tested 2 days later, under sober conditions.

**Results:**

As expected, AMP increased memory of studied words relative to placebo, and THC reduced memory of studied words. Although neither drug significantly affected false memory relative to placebo, AMP increased false memory relative to THC. Across participants, both drugs’ effects on true memory were positively correlated with their effects on false memory.

**Conclusions:**

Our results indicate that AMP and THC have opposing effects on true memory, and these effects appear to correspond to similar, albeit more subtle, effects on false memory. These findings are consistent with previous research using the DRM illusion and provide further evidence that psychoactive drugs can affect the encoding processes that ultimately result in the creation of false memories.

## Introduction

Memory plays a critical role in survival and must often be accurate. Memory is sometimes inaccurate, however, and can be so in predictable ways. One form of memory inaccuracy is false memory—i.e., the process of incorrectly remembering an event, or details of an event, that did not actually take place (Roediger 1996). A small number of studies suggest that psychoactive drugs may influence false memory, but the effects of these drugs on this phenomenon have not been examined in depth. Research into the effects of drugs on false memory is important because it both improves our understanding of the processes underlying the formation of false memories and contributes to our knowledge of the potential ways in which drugs can alter cognition and behavior.

False memory processes are commonly studied using the Deese/Roediger-McDermott (DRM) illusion (Deese 1959; Roediger and McDermott 1995). This task creates a robust memory illusion, and has provided key insights into the cognitive and neural processes that regulate false memory (for a review see Gallo 2010). In the DRM task, subjects study lists of words (e.g., bed, rest, awake, tired, dream) that are semantically associated to a non-presented word (e.g., sleep), referred to as the critical lure. These critical lures are often falsely remembered as having been presented in the study list, with false recall and false recognition rates significantly greater than baseline (unrelated lures). Unlike more traditional episodic memory tasks in which performance is highly accurate, the DRM task reliably elicits very high levels of false memories in cognitively normal participants, making it particularly useful for testing false memory theories.

Previous research into the effects of psychoactive drugs on DRM task performance provides some indication that drugs which alter true memory ability may also affect processes regulating false memory. However, most of this work has been limited to memory-impairing drugs, and the findings are somewhat inconsistent. Alcohol (Garfinkel et al. 2006; Milani and Curran 2000; Mintzer and Griffiths 2001b); the benzodiazepines triazolam (Mintzer and Griffiths 2000), diazepam, and lorazepam (Huron et al. 2001); and the anticholinergic drug scopolamine (Mintzer and Griffiths 2001a) have all been studied using similar versions of the DRM task (i.e., old/new recognition assessment). While each of these drugs significantly impaired memory for studied words, their false memory effects were more variable. For example, triazolam simultaneously reduced false recognition of critical lures and increased false recognition of unrelated lures (Mintzer and Griffiths 2000). Scopolamine’s profile resembled that of triazolam, in that it also reduced false recognition of critical lures; however, unlike triazolam, scopolamine did not significantly increase false recognition of unrelated lures (Mintzer and Griffiths 2001a). Lorazepam, on the other hand, significantly increased false recognition of unrelated lures, *without* affecting false recognition of critical lures (Huron et al. 2001). Neither alcohol nor diazepam had significant effects on false recognition of critical or unrelated lures at doses that impaired memory for studied words (Huron et al. 2001; Mintzer and Griffiths 2001b). Finally, a moderate dose of alcohol had no effect on critical lure false recognition in one study (Mintzer and Griffiths 2001b), but in another study, the same dose both decreased and increased critical lure false alarms when different versions of the task were used (e.g., stem completion, free recall, list repetition; Garfinkel et al. 2006). Thus, additional studies are necessary to determine how different memory-impairing drugs may differentially influence false memory processes.

Studying the effects of drugs on DRM task performance can provide valuable information regarding the cognitive processes that cause false memory. According to the activation/monitoring framework (e.g., Roediger et al. 2001), a critical lure is falsely remembered to the extent that: (1) it is mentally *activated* by associative processes and (2) the participant fails to correctly *monitor* the source of this activation, attributing it to actual presentation in the study list. By design, the associative relationship between studied list words and their critical lure enhances the likelihood that the critical lure will be activated at encoding, as well as the likelihood that this critical lure will feel familiar when it is encountered on a subsequent memory test. Activation therefore increases the likelihood that critical lures will be falsely remembered, relative to unrelated lures. Monitoring, on the other hand, refers to the process by which nonstudied items are correctly identified as new, as when participants use very conservative retrieval criteria and endorse only the strongest memories. To the extent that both related (critical) and unrelated lures are relatively weaker in memory than studied words, using a more conservative monitoring criterion can affect both kinds of lures (see Gallo 2006, for relevant research).

The activation/monitoring framework holds that drugs can impact false memory through actions on either associative activation or monitoring. As a result, drugs that impair memory for studied words might have opposing effects on false memory. On the one hand, decreased processing of the studied words should decrease activation of the related critical lure, thereby decreasing false memory. Such a profile is consistent with effects reported for scopolamine, triazolam, and, under certain conditions, for alcohol (Garfinkel et al. 2006; Mintzer and Griffiths 2000, 2001a). On the other hand, impaired memory for studied words could also correspond to a reduced ability to accurately monitor memory, thereby increasing false memory. Indeed, this would explain why triazolam and lorazepam were found to increase false recognition of unrelated lures (Huron et al. 2001; Mintzer and Griffiths 2000). Taken together, it appears as though drugs that impair true memory ability may impair associative activation, monitoring, or both. However, as a whole, these drugs have tended to reduce associative false recognition, suggesting a larger effect on activation than on monitoring.

If this interpretation of memory-impairing drug studies is correct, then drugs that enhance memory for studied words should enhance associative activation, monitoring, or both. To our knowledge, the only drug with memory-enhancing properties to be tested in the DRM task is the stimulant drug caffeine. Capek and Guenther (2009) found that a dose of caffeine that enhanced memory for studied list words also increased false recall of critical lures. These effects are consistent with the idea that caffeine enhanced false memory activation. Still, the use of a recall test limits a direct comparison to the aforementioned effects of memory-impairing drugs on recognition tests. For instance, a more liberal response criterion might have enhanced false recall, in the absence of an effect on activation processes. Because recall tests do not measure false recognition to unrelated lures, these effects are difficult to disentangle. Studies that directly compare cognition-enhancing and cognitive-impairing drugs, using the same testing procedures, are needed to resolve these questions.

In summary, there is some evidence that drugs that affect true memory may affect false memory in parallel, perhaps due to their effects on associative activation processes. However, the concordance between impairments of true memory and the incidence of false memory is inconsistent, and only one DRM study has examined a memory-enhancing drug. Further, in including both the study phase and the test phase during the same drug session, the majority of studies have failed to differentiate between drugs’ effects on specific stages of memory, as drugs may affect either encoding processes (i.e., initial perception and subsequent consolidation), retrieval processes, or both. We are aware of only one study that administered a psychoactive drug at encoding but tested participants in a separate session. Garfinkel et al. (2006) found that alcohol administered at the time of encoding reduced false memory when participants were tested days later while sober—results that contrast with those of Mintzer and Griffiths (2001b), who found no effect of alcohol on DRM false memory when participants were under the influence for both study and test phases. Collectively, these examples highlight the need for additional studies that separate the effects of drugs at specific stages of memory (e.g., encoding).

Here, we present the results of two parallel studies in which healthy human volunteers received either dextroamphetamine (AMP) or Δ^9^-tetrahydrocannabinol (THC) before studying DRM word lists, and had their memory tested in a drug-free state, 2 days later. We selected AMP because it enhances recall and recognition memory at moderate doses (Hurst et al. 1969; Mintzer and Griffiths 2003, 2007; Soetens et al. 1993, 1995; Zeeuws and Soetens 2007), and THC because it is known to impair recall and recognition memory (Leweke et al. 1998; Miller and Cornett 1978; Miller et al. 1977). As a result, we were able to compare two drugs with opposing effects on memory encoding on performance in the DRM task. Based on our interpretation of the studies described above, we expected that AMP would enhance encoding processes and associative activation, resulting in increased false recognition of critical lures. In contrast, THC would reduce encoding processes and associative activation, resulting in reduced false recognition of critical lures. An improved understanding of how drugs can affect false memory is important because it provides insight into basic memory processes. Further, given the widespread licit and illicit use of AMP and THC, effects of either drug on false memory may have significant clinical and social implications.

## Methods

### Subjects

Healthy volunteers, aged 18–35 years, were recruited from the University of Chicago and surrounding community via posters, advertisements, and word-of-mouth referrals, and participated either in the AMP study (*N*=25; 12 female) or the THC study (*N*=25; 11 female). The studies were conducted separately, but the same inclusion criteria and methods were used. All participants underwent an in-person psychiatric interview and physician-supervised physical examination including an electrocardiogram, and they completed a health questionnaire with detailed information on current and lifetime drug use. Exclusion criteria included current Axis I DSM-IV disorder including any substance dependence (American Psychiatric Association 1994) other than tobacco dependence. Volunteers were also excluded if they had a history of psychosis or mania, less than a high school education, lack of fluency in English, a body mass index outside of 19–26 kg/m^2^, high blood pressure (>140/90), an abnormal electrocardiogram, reported daily use of any medication other than birth control, or were pregnant, lactating, or planning to become pregnant in the next 3 months. For ethical reasons, participants were only eligible if they reported using cannabis more than 10 times in their lifetime with no serious cannabis-related adverse events. Inclusion was limited to individuals not currently using cannabis more than three times per week, to reduce the likelihood of tolerance to THC’s effects on memory (D’Souza et al. 2008). Women not taking hormonal contraceptives in the AMP group (*n*=6 of 12) were tested during the first 2 weeks of their menstrual cycles only because responses to the drug vary across the cycle (White et al. 2002).

### Design

Two parallel studies were carried out investigating the effects of AMP and THC, respectively, on true and false memory. Both studies utilized a three-session, double-blind, placebo-controlled, within-subjects design. Each of the three sessions consisted of two laboratory visits—a 4-h visit (encoding phase) followed exactly 2 days later by a 1-h visit (retrieval phase). During the encoding phase visits, participants received capsules containing placebo or AMP (10, 20 mg; AMP group) or THC (7.5, 15 mg; THC group) in random order, before viewing DRM word lists (see task description below). Memory for the studied words was assessed at the subsequent retrieval phase visit, during which no drugs were administered. These data were obtained as part of a larger study investigating the effects of AMP and THC on the processing of emotional stimuli. These other procedures were held constant with respect to the current procedure and will not be described here. This study was approved by the relevant University of Chicago institutional review board.

### Procedure

Qualifying participants attended a 1-h orientation session to explain the procedures and risks associated with the study, provide informed consent, and practice study tasks and questionnaires. They were informed that it was a study on the effects of drugs on mood and memory, and that they might receive a placebo, stimulant (e.g., amphetamine), sedative/tranquilizer (e.g., Valium), or a marijuana-like drug. They were instructed to consume their normal amounts of caffeine and nicotine before sessions, but to abstain from using alcohol, prescription, or over the counter drugs for 24 h before the session. They were told that they would be tested for drug use before each session to verify abstinence. Participants were also instructed to get their normal amounts of sleep, and not to eat solid food for 2 h before experimental sessions.

Participants were tested individually in comfortably furnished rooms with a television and VCR, magazines, and a computer for administering questionnaires and tasks. They were allowed to watch television, movies, or read when no measures were being obtained, but they were not allowed to sleep, work, or study, and they had no access to cell phones or internet. Upon arrival for all six visits, participants first completed compliance measures including breath alcohol levels (Alco-sensor III; Intoximeters, St. Louis, MO) and urine drug (ToxCup; Branan Medical Co., Irvine, CA), and pregnancy tests (women only, using an hCG assay; Aimstrip; Craig Medical, Vista, CA). During the encoding phase visits, after completing pre-capsule subjective ratings and physiological measures (see “Dependent measures” below), participants subsequently ingested capsules (0 min) containing placebo or active drug with 100 mL water. For the remainder of the encoding phase visits, participants completed subjective mood and drug effect ratings and provided cardiovascular measurements every 30 min. At 135 min after capsule ingestion, participants studied DRM list words. Drug effects were still expected to be present at this time (Wachtel et al. 2002; White et al. 2007). At the end of the encoding phase visits, participants completed an end-of-session questionnaire and were allowed to leave after residual subjective and physiological drug effects subsided. During the retrieval phase visits, participants returned to the laboratory for 1 h to complete mood questionnaires and the recognition memory task. All participants were fully debriefed at study completion.

### Dependent measures

#### Memory task

False memory was measured with the Deese/Roediger–McDermott illusion (Deese 1959; Roediger and McDermott 1995). Participants studied lists of semantically associated words, and were later instructed to correctly discriminate studied list words from nonstudied words. The nonstudied words included both those that were semantically closely related (critical lures) or unrelated (unrelated lures) to the studied list words. When memory for the studied words is tested, there is a strong tendency for subjects to incorrectly identify critical lures as having been previously studied. This tendency to erroneously recognize the critical lures, relative unrelated lures, is the measure of false memory. This task provides a measure of true memory for studied words and false memory for both related and unrelated words. The stimuli consisted of 30 lists of 10 words each drawn from the false memory norms provided by Roediger et al. (2001), and an additional 30 words drawn from lists that were not used in this experiment (critical lures and list words) to provide unrelated lures (10 unrelated lures per session).

##### Encoding phase

During the encoding phase visits, participants viewed 10 lists of 10 words each. Different sets of lists were used in each of the three encoding phase visits, but the words within the lists were always presented in order of descending associative strength with the critical lure. Words were displayed individually on a computer screen for 3,000 ms each. The participant initiated the presentation of each word by pressing a key. To ensure that participants would meaningfully process the studied words under each of the drug conditions, they were required to make a pleasantness judgment (pleasant/unpleasant) for each word using the keyboard. Prior research indicates that pleasantness judgments afford meaningful semantic processing of studied words (Craik and Tulving 1975), which can enhance the false recognition illusion relative to more shallow levels of processing (e.g., Thapar and McDermott 2001). Pleasantness ratings were self-paced.

##### Retrieval phase

Two days after each encoding phase visit, participants completed a retrieval phase, during which their recognition memory was assessed. Recognition test lists consisted of 30 words: 10 studied words (first word presented from each list during the respective encoding phase), the 10 critical lures that were associated to these study lists but were not themselves studied, and 10 unrelated lures. The order of the words was randomized, but they were otherwise presented in the same manner as during the encoding phase. Participants were instructed to identify words they had seen on the previous encoding session (yes/no). Raw outcome measures included hit rate (the proportion of studied words correctly recognized at test), false alarm rate for critical lures (the proportion of critical words incorrectly recognized at test), and false alarm rate for unrelated lures. Main outcome measures included “adjusted true memory” (hit rate minus unrelated lure false alarm rate; scale, −100 to 100) and “adjusted false memory” (critical lure false alarm rate minus unrelated lure false alarm rate; scale, −100 to 100). This method of subtracting unrelated lure false alarms from hits and critical lure false alarms is widely used in order to control for changes in base rate responding (Gallo 2006).

#### Subjective effects measures

Participants completed several subjective effect ratings on each visit (Ballard et al. in review), but for the purpose of this analysis, we examined only their ratings of subjective stimulation [visual analog ratings of feeling “stimulated” (Folstein and Luria 1973), rated from 0 to 10], ratings of overall drug effect [Drug Effects Questionnaire (Fischman and Foltin 1991); DEQ “feel” drug rated from 0 to 10], and drug preference (calculated as DEQ ratings of “like” the drug effect minus DEQ ratings of “dislike” the drug effect; possible range −10 to +10). We calculated each subject’s mean change-from-pre-capsule baseline scores assessed immediately before and after DRM list studying (+120 and +150 min post-capsule) to obtain a summary measure of subjective drug effect.

#### Physiologic effects measures

Blood pressure (BP) and heart rate (HR) were measured at baseline, and every 30 min post-capsule, using a portable digital blood pressure monitor (AND Medical/Life Source, San Jose, CA). Mean change-from-pre-capsule baseline scores assessed immediately before and after DRM list studying (+135 and +165 min post-capsule) were calculated and examined in relation to effects of the study drugs on memory.

### Drugs

AMP (Barr Laboratories, Pomona, NY) and THC (Marinol® [dronabinol]; Solvay Pharmaceuticals, Marietta, Georgia) were placed in opaque size 00 capsules in doses of 10 or 20 mg (AMP) or 7.5 or 15 mg (THC), with dextrose filler. Placebo capsules contained only dextrose. Capsules were administered in counterbalanced order under double-blind conditions. These doses of AMP and THC are within the range shown to affect memory in previous studies (Curran et al. 2002; Leweke et al. 1998; Soetens et al. 1995; Zeeuws and Soetens 2007), and the oral route ensures a steady time course of response throughout the study session (Wachtel and de Wit 2000; White et al. 2006).

### Statistical analyses

#### Memory task

We first confirmed that the two groups did not differ on critical demographic variables. Then, two-way ANOVAs were conducted to determine whether AMP and THC differed in their effects on each of the five memory measure outcomes with drug treatment group (drug type) as the between-subjects factor, and drug dose (dose) as the within-subject repeated measure. Results were satisfactory for model assumptions of normality (Shapiro–Wilk test *p*>0.01) and homogeneity of covariance (Box’s test *p*>0.01). For all other analyses, alpha was set at *p*=0.05, and partial eta-squared values are included where appropriate as a measure of effect size. We also examined the effects on memory of each drug individually using similar one-way ANOVAs. Significant main effects of dose (*p*<0.05) were followed up by post hoc paired *t* tests to test the dose dependency of the effect, and Cohen’s *d*s are included as a measure of effect size where appropriate. Where violations of sphericity were apparent in ANOVAs (*p*<0.05), Greenhouse–Geisser corrected results are presented.

#### Subjective and physiologic effects of AMP and THC at encoding, and relation to memory performance

Drug effects on subjective and physiologic state were analyzed using repeated-measures ANOVAs, with drug type (AMP or THC) as the between-subject factor and dose as the within-subject factor. For each participant, we calculated the mean of the change-from-pre-capsule scores obtained immediately before and after the participants studied DRM lists. Significant main effects of drug dose were followed by paired *t* tests, and Greenhouse–Geisser corrections were applied as necessary. We performed exploratory analyses to examine relationships between the drugs’ effects drug on memory and their effects on subjective and physiologic state. Bivariate correlational analyses of drug effect (drug minus placebo) were conducted on these measures for each dose of each drug separately. Alpha was set liberally at *p*=0.05 for correlational analyses, given the exploratory nature of these analyses and to facilitate generation of novel hypotheses for future investigation.

## Results

### Participant characteristics

The demographic characteristics of the participants in the AMP (*N*=25; 12 female) and THC (*N*=25; 11 female) studies are summarized in Table 1, and lifetime recreational drug use histories are summarized in Table 2. All participants were current or past occasional recreational cannabis users. Most were in their early twenties, Caucasian, and reported light to moderate use of alcohol and other drugs. The groups did not differ on any of the demographic characteristics or on performance in the memory tasks (below) under placebo conditions.

### Effects of AMP and THC on DRM task performance

The raw hit rates and false alarm rates for each item type are presented in Table 3. As expected, under placebo conditions, participants in both groups accurately discriminated studied list words from critical and unrelated lures, and critical lures were more likely than unrelated lures to be falsely recognized as studied (all *p*s <0.01). The two groups did not differ in DRM performance measures on the placebo sessions, and performance did not vary as a function of session number (analyses not included). To investigate the drug effects on true and false memory, we next report analyses of the true and false memory scores, using the adjustment procedure described in “Methods”.

#### True memory

**Fig. 1:**
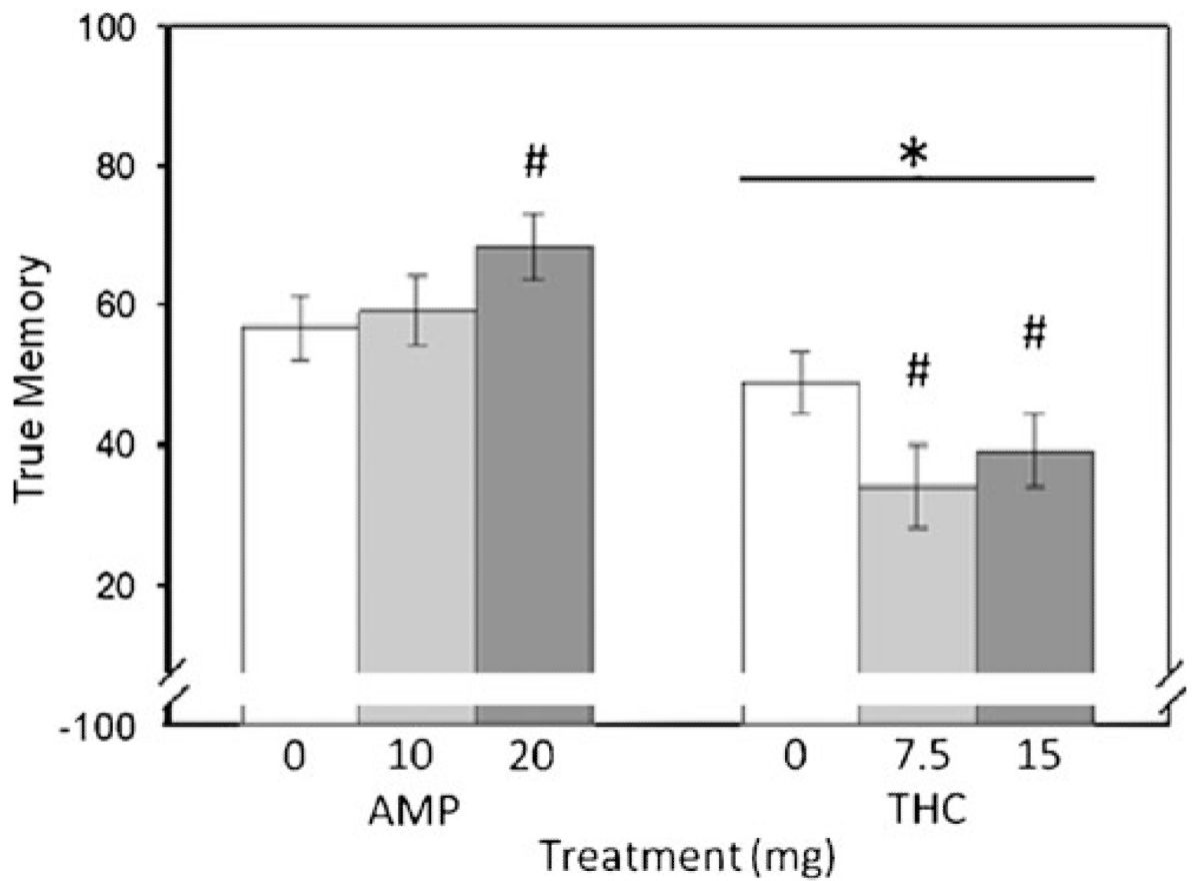
Effects of AMP and THC on true memory. *p<0.05; significant main effect of drug type. #*p*<0.05 compared to respective placebo condition; one-tailed *t* test

Consistent with our predictions, there were large true memory differences between the AMP and THC conditions (Fig. 1). AMP improved accurate memory relative to THC, as evidenced by a significant main effect of drug type (*F*[1, 48]=16.69, MSE=0.097, *p*<0.001, partial *η*^2^=0.26).^1^ Accordingly, the two groups differed significantly on true memory after both active drug doses (both *p*s <0.01), but they did not differ under placebo conditions (*p*=0.2), and there was a significant dose × drug type interaction (*F*[2, 96]=3.51, MSE=0.045, *p*=0.034, partial *η*^2^=0.07). Although follow-up *t* tests comparing each dose of each drug to placebo failed to reach significance, we used a more liberal threshold (one tailed) because of our strong a priori predictions for drug effects on true memory. These analyses revealed that AMP enhanced true memory relative to placebo at the higher dose (placebo vs 10 mg: n.s.; placebo vs 20 mg: *t*[24]=1.98, *p*=0.03, *d*= 0.5; one tailed), and THC impaired true memory at both doses (placebo vs 7.5 mg: *t*[24]=2.35, *p*=0.014, *d*=0.56; placebo vs 15 mg: *t*[24]=1.75, *p*=0.047, *d*=0.4; one tailed).

#### False memory

**Fig. 2:**
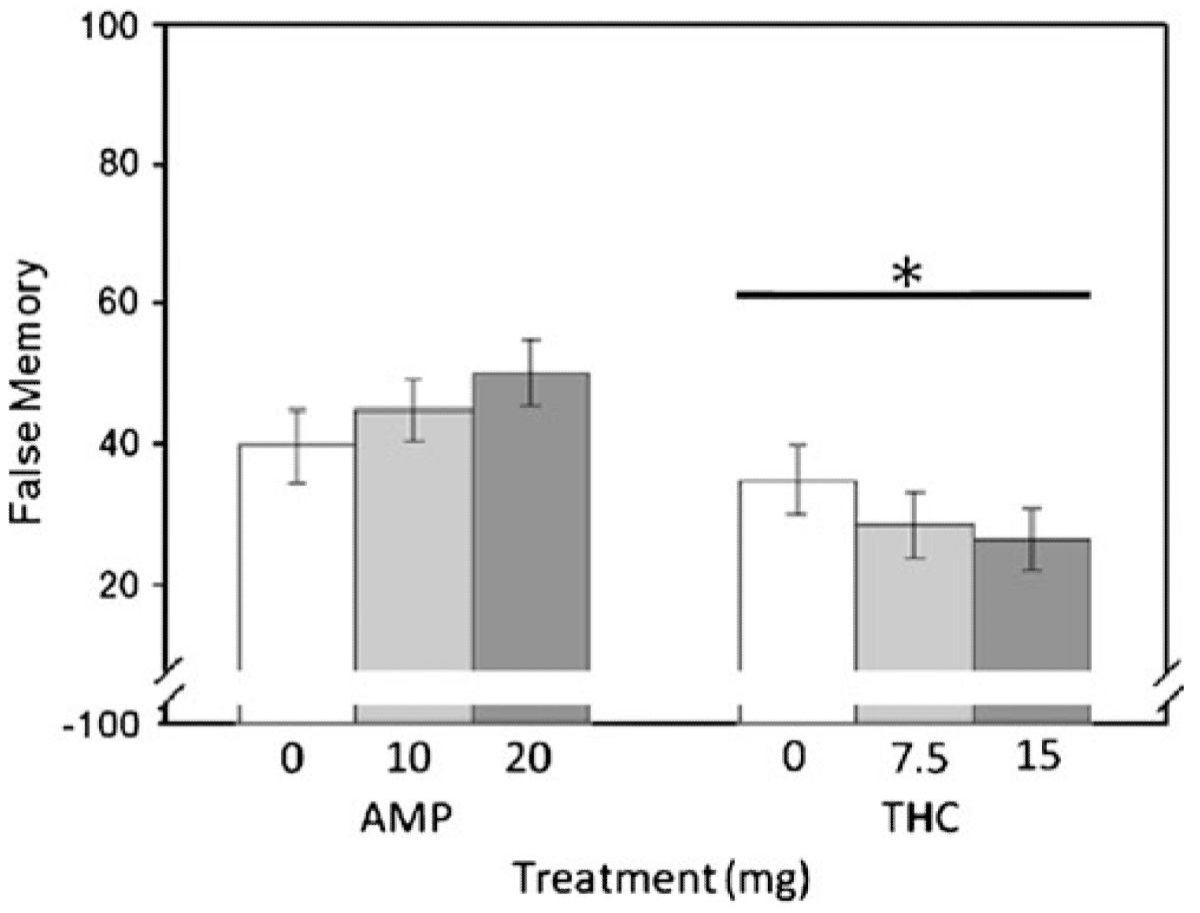
Effects of AMP and THC on false memory. *p<0.05; significant main effect of drug type

Inspection of Fig. 2 suggests that the effects of the drugs on false memory parallel those seen for true memory. Consistent with true memory findings, AMP increased false memory relative to THC (*F*[1, 48]=14.70, MSE=0.057, *p*<0.001, partial *η*^2^=0.234).^2^ While the false memory dose × drug type interaction failed to reach significance (*F*[2, 96]=2.07, *p*=0.132), as was the case with true memory, the two treatment groups differed significantly from one another on false memory in both drug-dose conditions (*p*s ≤0.01), but not in the placebo condition (*p*=0.5). However, in contrast to effects on true memory, none of the drug-dose conditions were significantly different from the relevant placebo condition on false memory (*p*s ≥0.17; two tailed).

#### Relating true and false memory

**Fig. 3:**
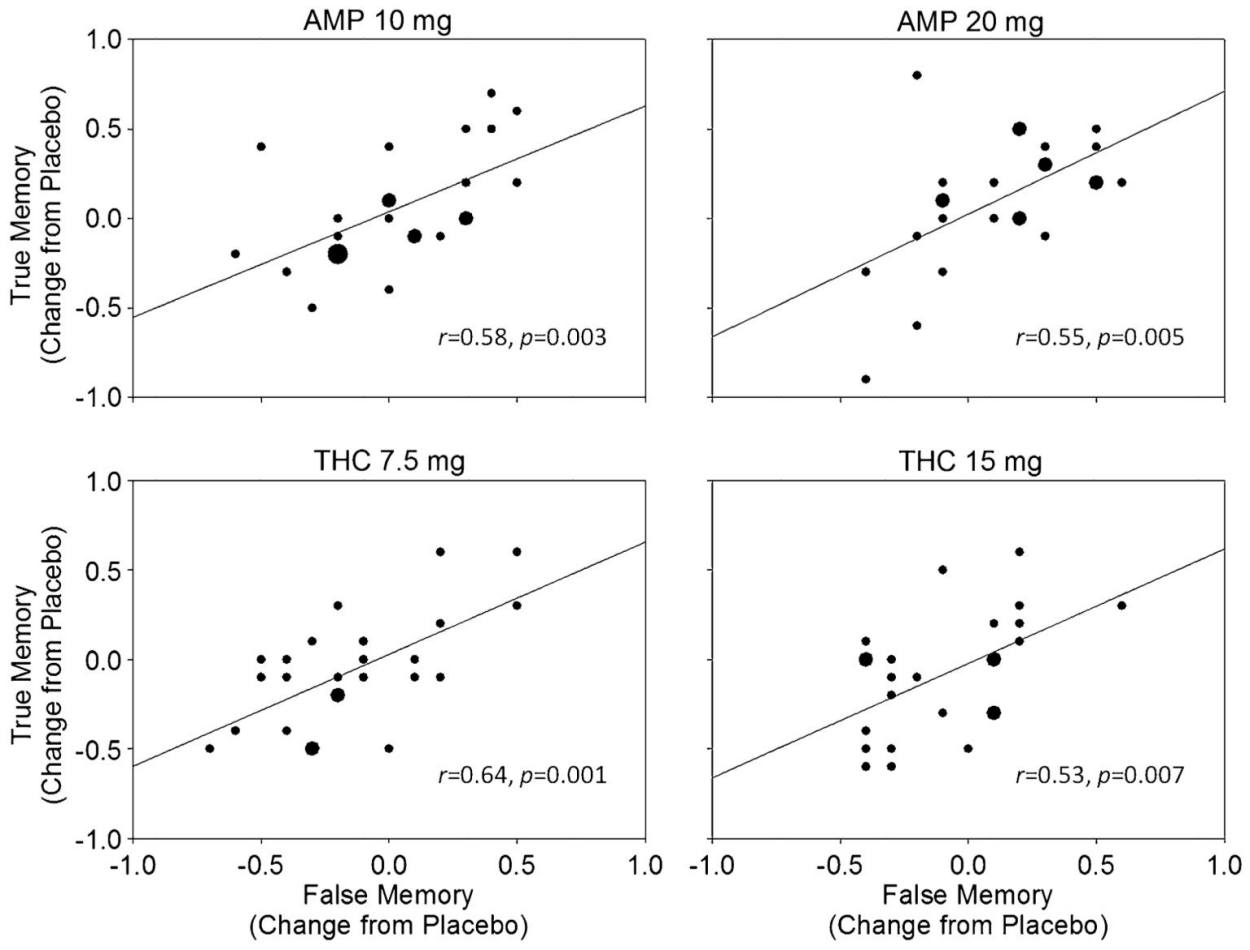
Drug-induced changes in true memory were positively correlated with changes in false memory for both AMP and THC

To further investigate the potential relationship between drug-induced true and false memory changes, we performed correlational analyses using every participant’s change-from-placebo true and false memory scores calculated for each dose of each drug individually (Fig. 3). The first point to take from these figures is that each drug had variable effects on both true and false memory across individuals, with some individuals responding positively to each drug (i.e., greater true and false memory relative to placebo) and some individuals responding negatively to each drug (i.e., reduced true and false memory relative to placebo). This individual variability might explain why the overall effects of each drug on true and false memory were rather weak relative to placebo. Also note that individuals generally responded more positively to AMP than to THC, for both true and false memory, consistent with predictions and with the significant drug effects found when directly comparing these two drugs.

The second point to take from these figures is that there were strong positive correlations between true and false memory across individuals, at both doses of each drug (AMP: *r*[23]=0.58, *p*=0.003 and *r*[23]=0.55, *p*=0.005 for 10 and 20 mg, respectively; THC: *r*[23]=0.64, *p*=0.001 and *r*[23]=0.53, *p*=0.007 for 7.5 and 15 mg, respectively). Thus, greater drug-induced true memory changes corresponded to greater changes in false memory. These analyses provide additional evidence that the effects of either drug on associative false memory were analogous to their effects on true memory, lending further support to the associative activation hypothesis.

### Subjective and physiologic effects of AMP and THC at encoding, and relation to memory performance

The drugs’ effects on mood and physiology at the time DRM lists were encoded are summarized in Table 4. Participants reported feeling the effects of both drugs, but they rated feeling both doses of THC more strongly than either dose of AMP. In contrast, the 20 mg dose of AMP produced the greatest subjective stimulation, followed by both doses of THC. Participants indicated a preference for AMP (20 mg only; calculated as “dislike drug effects” scores subtracted from “like drug effects” scores) but not for THC. Accordingly, there was wide interindividual variability in preference for THC’s effects (range −5.7 to 6.55 and −8.65 to 5.9, for the 7.5 and 15 mg dose, respectively). Further, only 15 of the 25 study participants indicated a desire to take the 7.5 mg dose of THC again, and only 11 of the 25 reported a desire to take the 15 mg dose of THC again. Despite the modest subjective effects, both doses of AMP significantly increased physiological stimulation measures, including systolic and diastolic BP, and heart rate. By comparison, THC’s effects on physiologic stimulation were smaller, and significant increases were seen only on diastolic BP and heart rate at the 15 mg dose. Exploratory analyses suggested that the effects of the drugs on memory performance were largely independent of their subjective and physiological effects.

## Discussion

We examined the effects of acute AMP and THC, two drugs with opposing influences on memory, on false memory using the DRM illusion. As expected, AMP tended to increase, whereas THC tended to reduce, memory for studied list words. Analogous trends were also seen for false memory. While these effects on false memory did not reach statistical significance for either drug relative to placebo, the two drugs differed significantly in false memory when directly compared to each other, such that false memory was greater in the AMP conditions than in the THC conditions. We also observed strong positive correlations between drug-induced true and false memory changes across individuals, at each dose of each drug. As a whole, these results indicate that the effects of psychoactive drugs on true memory (be they positive or negative) tend to have parallel effects on associative false memory.

These parallel effects on true and false memory are broadly consistent with previous drug studies (Capek and Guenther 2009; Garfinkel et al. 2006; Mintzer and Griffiths 2000, 2001a), and extend these patterns to a situation where both a memory-impairing and a memory-enhancing drug could be evaluated in a single experimental paradigm. Our studies had the further strength of assessing the drugs’ effects specifically on the memory encoding phase. The encoding phase occurred during peak drug effects, and the participants were tested on a separate day, in a sober state. This aspect of our design is theoretically important. As discussed by Mintzer and Griffiths (2000), drugs that have parallel effects on true and false memory resemble the effects of organic amnesia, such as that attributed to dysfunction of the hippocampus and surrounding structures. Not only are individuals with organic amnesia less likely to correctly recognize studied information in the DRM task, but they also are less susceptible to false recognition of critical lures (Schacter et al. 1996). Similar to the effects of memory-impairing drugs, these findings suggest that such amnesia impairs the false memory associative activation process, potentially by interfering with the ability to encode associations that would subsequently lead to critical lure false recognition. However, this type of brain damage also is likely to affect retrieval processes, making the relative contribution of encoding and retrieval processes difficult to disentangle (for review discussion, see Gallo 2006). In confining our drug effects to the encoding phase, our results more clearly indicate that the associative activation process can be affected by encoding manipulations alone.

Our individual difference analyses may shed light on prior inconsistencies in drug effects on DRM false memory. We, like some previous studies (Huron et al. 2001; Mintzer and Griffiths 2001b), failed to detect significant drug effects on DRM false memory relative to placebo with doses of drugs that altered true memory ability. Although both AMP and THC had moderate and opposing effects on true memory overall, the correlational analyses revealed a more complex profile. These analyses provide some suggestion that the relatively weak effects of AMP and THC on true memory at the group level were due, at least in part, to variability at the individual level. Surprisingly, both drugs enhanced memory relative to placebo in some individuals and impaired memory relative to placebo in other individuals. As such, this variability in the drugs’ effects on true memory may have limited our ability to detect secondary, more moderate effects on false memory. Future work will be needed to determine whether this relationship holds for other drugs, but the fact that we observed it in two drugs with very different cognitive profiles suggests that it may be a general phenomenon.

These studies are the first to investigate the effects of either AMP or THC on false memory. As both of these drugs are widely used for medicinal and recreational purposes, it is important to understand how they might affect false memory processes. Our data indicate that these drugs can affect false memory in ways that relate to their effects on true memory, and also that individuals appear to differ considerably in how their memory is impacted by these drugs. This individual variability suggests that caution must be used when drawing conclusions about the effects of these drugs on memory accuracy. Future studies should investigate the sources of these individual differences and the generalizability of these effects to other kinds of false memory.

## Figures and Tables

**Table 1:** Participants’ demographics and recent drug use

	AMP group			THC group		
	Mean	SD	Range	Mean	SD	Range
Age (years)	24.00	3.65	18–33	24.36	4.56	18–35
Body mass index	23.02	1.93	19–26	22.45	2.04	19–26
Education (years)	15.36	1.50	12–18	15.48	1.45	12–18
Caffeine (cups/day)	1.43	0.98	0–4	1.64	1.08	0–5
Nicotine (cigarettes/week)	20.76	23.67	0–80	14.76	25.83	0–105
Alcohol (drinks/week)	10.10	7.93	2–30	7.12	4.20	0–14
Cannabis (times/month)	2.66	3.73	0–12	2.12	2.54	0–8

**Table 2:** Participants’ lifetime occasions of recreational drug use

	AMP group (*n*)					THC group (*n*)				
	Never	1–10	11–50	51–100	100+	Never	1–10	11–50	51–100	100+
Cannabis	0	1	7	6	11	0	0	10	1	14
Sedatives^a^	21	4	0	0	0	17	5	2	1	0
Stimulants^b^	14	7	3	0	1	10	5	5	2	3
Opioids^c^	20	5	0	0	0	9	9	4	3	0
Hallucinogens^d^	10	14	1	0	0	10	8	4	0	3
Inhalants^e^	23	2	0	0	0	18	5	2	0	0

a e.g., diazepam, alprazolam, clonazepam

b e.g., amphetamine, methylphenidate, cocaine, methamphetamine

c e.g., codeine, morphine, oxycodone, heroin, opium

d e.g., lysergic acid diethylamide, psilocybin, mescaline, ±3,4-methylenedioxymethamphetamine

e e.g., nitrous oxide, amyl nitrite

**Table 3:** Raw proportions of responses ±SEM

Drug		Hit rate	Critical lure FA rate	Unrelated lure FA rate
AMP	Placebo	0.78±0.03	0.61±0.04	0.22±0.03
	10 mg	0.76±0.05	0.61±0.05	0.16±0.04
	20 mg	0.83±0.04	0.64±0.05	0.14±0.03
THC	Placebo	0.72±0.03	0.58±0.04	0.23±0.04
	7.5 mg	0.61±0.05	0.56±0.05	0.27±0.04
	15 mg	0.63±0.04	0.50±0.05	0.24±0.04

**Table 4:** Effects of AMP and THC on select mood and physiological measures at the time of DRM list studying

	AMP group				THC group			
	Placebo	10 mg^a^	20 mg^a^	Dose^b^ (*F*_2, 23_)	Placebo	7.5 mg^a^	15 mg^a^	Dose^b^ (*F*_2, 23_)
Feel—DEQ (range 0–10)	0.32 (0.10)	0.84 (0.35)	1.88* (0.46)	**8.80**	0.14 (0.06)	2.63* (0.53)	3.53* (0.58)	**22.58**
Drug Preference (range −10 to 10)	0.02 (0.16)	0.81 (0.36)	2.63* (0.67)	**12.67**	−0.03 (0.04)	0.31 (0.57)	−0.91 (0.75)	1.58
Stimulated—VAS (range 0–10)	−0.89 (0.23)	−0.24 (0.36)	1.48* (0.56)	**14.62**	−0.54 (0.17)	0.90* (0.42)	0.78* (0.49)	**6.00**
BP (systolic)	−5.42 (1.52)	5.64* (0.97)	10.02* (2.35)	**18.00**	−4.08 (1.25)	−1.78 (2.00)	−0.10 (1.73)	1.61
BP (diastolic)	−2.72 (1.30)	5.96* (1.00)	12.66* (1.54)	**32.21**	−2.28 (1.14)	−1.40 (1.11)	1.76* (1.54)	**4.08**
HR	−9.74 (1.65)	2.86* (1.67)	4.92* (1.80)	**26.92**	−7.44 (1.84)	−3.20 (1.84)	4.46* (2.77)	**10.84**

Data are change from baseline means (SEM) of ratings obtained immediately before and after DRM list studying (+120 and +150 min post-capsule). Bold values indicate a significant main effect of drug dose (*p*<0.05)

* *p* <0.05

a Two-tailed paired *t* tests—compared to placebo

b Repeated measures ANOVA

## Footnotes

1 Reanalysis of true memory data using change-from-placebo scores confirmed a significant effect of drug type (*p*=0.011).

2 Reanalysis of false memory data using change-from-placebo scores approached significance for an effect of drug type (*p*=0.07).
